# Wide area digital dermoscopy applied to basal cell carcinoma^☆^^☆☆^

**DOI:** 10.1016/j.abd.2019.08.030

**Published:** 2020-03-20

**Authors:** Gerson Dellatorre, Guilherme Augusto Gadens

**Affiliations:** Department of Dermatology, Hospital Santa Casa de Misericórdia de Curitiba, Curitiba, PR, Brazil

**Keywords:** Carcinoma, basal cell, Dermoscopy, Image processing, computer-assisted

## Abstract

In situations in when a dermoscopic record of a large lesion is desirable, the resulting images are usually restricted to a small field of view due to the limited diameter of dermatoscope lenses. This limitation often produces several photographs separately, thus losing the possibility of a single-image global evaluation. In these case reports, we show examples of a recently published image montage technique called Wide Area Digital Dermoscopy, in this case, applied to basal cell carcinomas.

## Wide area digital dermoscopy methodology

To create a WADD image, a standard dermoscope (10× magnification, polarized) attached to a digital camera and a computer with Adobe Photoshop CC software (Adobe Systems Incorporated, San Jose, CA, USA, v19.1.6) were used.

The following steps were performed:

1) Image acquisition: During this step, all the lesion was covered, performing a peripheral overlap in each photo (20–30% of the previous image area in each new picture).
2) Merging images: In Photoshop software, File Menu, Automate and Photomerge options were selected. In Layout options, Reposition was selected and acquired dermoscopic images were added.
3) Image viewing: A conventional built-in Windows or Mac image visualization software was used for image visualization.

## Case 1

**Figure 1:**
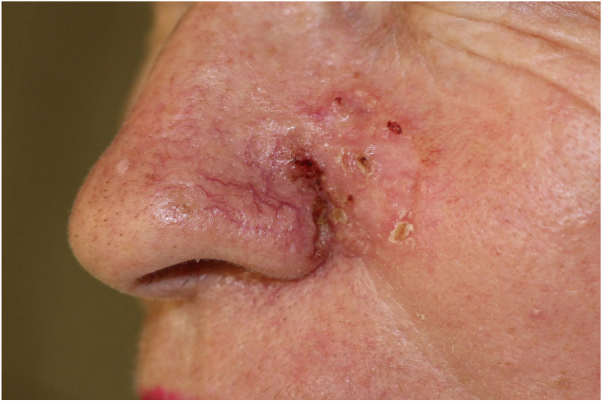
BCC. A poorly delimited lesion measuring 30 × 30 mm compromising the left cheek, lateral nasal wall and nasal ala.

**Figure 2:**
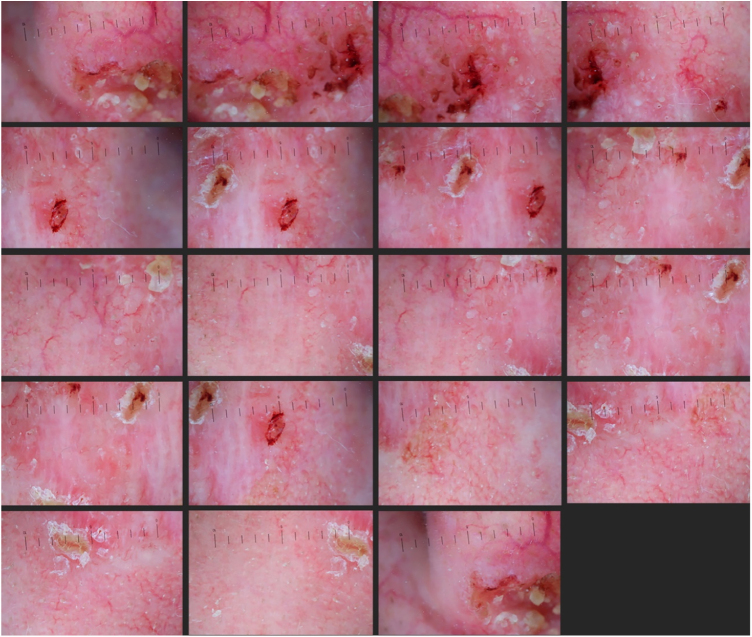
Nineteen overlapped dermoscopic images of the lesion (Heine Delta 20T – HEINE Optotechnik GmbH & Co, Herrsching, Germany) attached to a DSLR camera (polarized, ×10).

**Figure 3:**
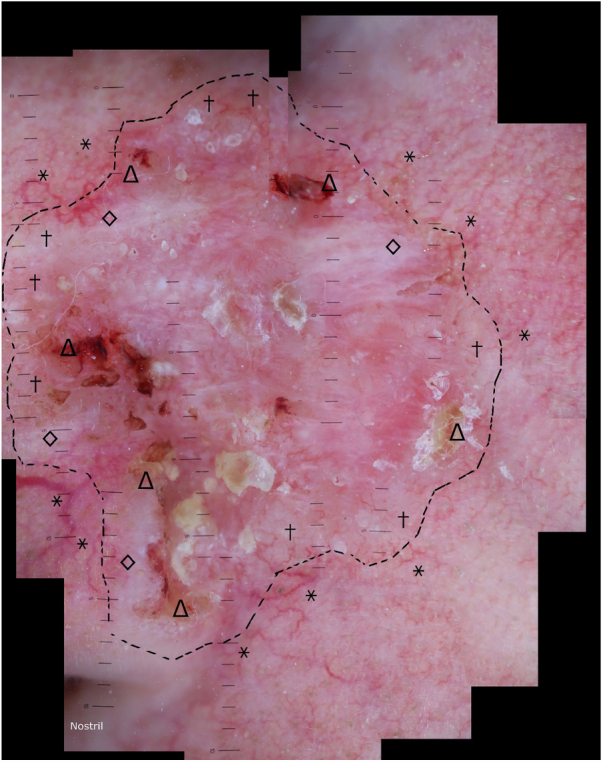
WADD image of BCC. Dermoscopic findings on the margins consisting of ulceration and crusts (Δ), cicatricial whitish areas (◊) and arboriform telangiectasias (†), in contrast to telangiectasias of the peripheral photodamaged skin (*).

56-year-old patient presented with a poorly delimited (Fig. 1), mixed type (superficial, nodular and infiltrative) basal cell carcinoma (BCC). After the acquisition of nineteen dermoscopic images with an overlap of 30% (Fig. 2), we merged them with Adobe Photoshop software (Adobe Systems Incorporated, San Jose, CA, USA, v19.1.6) by using its automatized “Photomerge” function, as previously detailed.^1^ The final WADD image obtained represented the full dermoscopic view from the lesional and perilesional areas (Fig. 3). The patient underwent Mohs Micrographic Surgery (MMS) for tumor excision, and margins were cleared after two stages due to a deep positive margin in the first stage.

## Case 2

**Figure 4:**
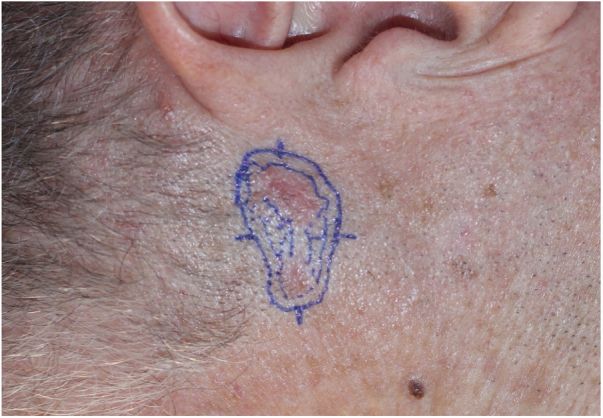
Basal cell carcinoma. A poorly delimited and noncontiguous lesion measuring 20 × 28 mm on the left preauricular area.

**Figure 5:**
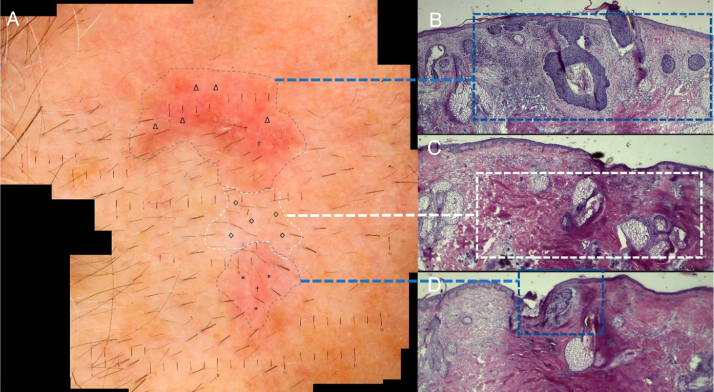
A, WADD image of BCC. B, Nodular and superficial BCC corresponding to erythematous structureless areas (Δ) and arboriform telangiectasias (†) on dermoscopy. C, Dermal fibrosis corresponding to a cicatricial-whitish area (◊), probably related to his previous topical treatment. D, Superficial BCC and dermal fibrosis corresponding to a milky-red area (*) and arboriform telangiectasias (†): (Hematoxylin & eosin x40).

A 55-year-old patient presented with a poorly delimited superficial and nodular BCC on his left preauricular area (Fig. 4). He had a history of topical 5-fluorouracil treatment on his face one year ago. We acquired sixteen separated dermoscopic images and merged them in the same way as previously mentioned. A dermoscopic-histologic correlation was made during MMS by analyzing frozen sections of tumoral debulking (Fig. 5). In this case, margins were cleared after one surgical stage.

## Discussion

Dermatologists can use WADD as a new register technique in digital dermoscopy. This tool can be useful in situations in which the limited field of view of the dermatoscope lenses difficult this register.^1^ For example, the use of WADD in the cases presented can serve as a didactic subsidiary in the teaching of tumoral margins demarcation and also in dermoscopic-histopathological correlations. The application of the method can still be beneficial in the dermatoscopic follow-up of more extensive melanocytic lesions, such as congenital melanocytic nevi, allowing an overall assessment of the lesions in their follow-up. Also, in trichology, extensive and unified registration of an affected hairy area would be useful for its detailed global follow-up.

The acquisition phase of the separate dermatoscopic images is a critical point in the process of creating a WADD image. The need for photographs to be obtained with a 30% overlap (a crucial technical factor for the software to recognize the melting points of images) can be challenging at the beginning of the practice, especially in large lesions. Another limiting factor of its use is the need for professional software (Photoshop) for the composition of the WADD image. In the future, the development of software with a user-friendly interface built-in on the imaging equipment itself may replace this phase of the compositing process.

## Financial support

None declared.

## Authors’ contributions

Gerson Dellatorre: Approval of the final version of the manuscript; conception and planning of the study; elaboration and writing of the manuscript; obtaining, analysis, and interpretation of the data; effective participation in research orientation; intellectual participation in the propaedeutic and/or therapeutic conduct of the studied cases; critical review of the literature; critical review of the manuscript.

Guilherme Augusto Gadens: Approval of the final version of the manuscript; conception and planning of the study; intellectual participation in the propaedeutic and/or therapeutic conduct of the studied cases; critical review of the literature; critical review of the manuscript.

## Conflicts of interest

None declared.
